# Probing Cocaine-Antibody Interactions in Buffer and Human Serum

**DOI:** 10.1371/journal.pone.0040518

**Published:** 2012-07-10

**Authors:** Muthu Ramakrishnan, Fernando Alves De Melo, Berma M. Kinsey, John E. Ladbury, Thomas R. Kosten, Frank M. Orson

**Affiliations:** 1 Veterans Affairs Medical Center, Houston, Texas, United States of America; 2 Department of Internal Medicine, Baylor College of Medicine, Houston, Texas, United States of America; 3 Department of Pathology, Baylor College of Medicine, Houston, Texas, United States of America; 4 Department of Immunology Allergy and Rheumatology, Baylor College of Medicine, Houston, Texas, United States of America; 5 Department of Psychiatry, Baylor College of Medicine, Houston, Texas, United States of America; 6 Department of Biochemistry and Molecular Biology, MD Anderson Cancer Center, University of Texas, Houston, Texas, United States of America; 7 Center for Biomolecular Structure and Function, MD Anderson Cancer Center, University of Texas, Houston, Texas, United States of America; Concordia University Wisconsin, United States of America

## Abstract

**Background:**

Despite progress in cocaine immunotherapy, the kinetic and thermodynamic properties of antibodies which bind to cocaine and its metabolites are not well understood. It is also not clear how the interactions between them differ in a complex matrix such as the serum present in the human body. In the present study, we have used microscale thermophoresis (MST), isothermal titration calorimetry (ITC), and surface plasmon resonance (SPR) we have evaluated the affinity properties of a representative mouse monoclonal (mAb08) as well as those of polyclonal antibodies purified from vaccinated mouse and human patient serum.

**Results:**

MST analysis of fluorescently tagged mAb08 binding to cocaine reveals an approximately 15 fold decrease in its equilibrium dissociation constant in 20–50% human serum compared with that in saline buffer. A similar trend was also found using enriched polyclonal antibodies purified from vaccinated mice and patient serum, for which we have used fluorescently tagged bovine serum albumin conjugated to succinyl norcocaine (BSA-SNC). This conjugate closely mimics both cocaine and the hapten used to raise these antibodies. The ITC data also revealed that cocaine has a moderate affinity of about 2 µM to 20% human serum and very little interaction with human serum albumin or nonspecific human IgG at that concentration range. In a SPR inhibition experiment, the binding of mAb08 to immobilized BSA-SNC was inhibited by cocaine and benzoylecgonine in a highly competitive manner, whereas the purified polyclonal antibodies from vaccinated humans and mice, revealed preferential selectivity to pharmacologically active cocaine but not to the inactive metabolite benzoylecgonine. We have also developed a simple binding model to simulate the challenges associated with cocaine immunotherapy using the variable quantitative and kinetic properties of the antibodies.

**Conclusions:**

High sensitivity calorimetric determination of antibody binding to cocaine and its metabolites provide valuable information for characterization of their interactions and thermodynamic properties. In addition MST measurements of antibody affinity in the presence of biological fluids will provide a better opportunity to make reliable decisions and facilitate the design of cocaine vaccines and immunization conditions. The methods should be more widely adopted in characterization of antibody complexes.

## Introduction

Cocaine addiction continues to be a source of healthcare and socioeconomic problems throughout the world. Recent government surveys indicate that 2.4 million or more Americans aged 12 and older are addicted to cocaine [1], [2]. Despite enormous efforts from basic and clinical studies, the current treatments and medications are still not sufficiently effective in reducing cocaine addiction [3], [4]. Since the site of pharmacological effect is inside the brain, it was hypothesized that effective blockade of cocaine entry to the brain could be attained by having high affinity anti-cocaine antibodies in the peripheral blood circulation [1], [5]. This triggered the field of immunotherapy, including both passive administration of monoclonal antibodies [6], [7] and active stimulation of the patient’s immune system with conjugate vaccines to produce cocaine-specific endogenous antibodies [8]–[10]. The first step in either approach involves the covalent linkage of a nonimmunogenic cocaine derivative to an immunogenic carrier protein. Vaccination with this construct will provoke the immune system to produce antibodies specific to cocaine. It is desirable that these antibodies will primarily recognize cocaine and also the pharmacologically active derivative cocaethylene (CE, which is produced by a trans-esterification of cocaine with ethanol) in the blood stream. Screening monoclonal antibodies (mAb) provide an opportunity to select the optimum antibody in terms of selectivity and affinity from different polyclonal pools. However, in developing vaccines such screening options are not possible, and this issue needs to be addressed by fine tuning the hapten construction, linker, carrier proteins and adjuvants. Nevertheless, despite these challenges great progress has been made recently in translating cocaine immunotherapy to advanced clinical trials. However, our understanding of the interactions between cocaine and the antibodies produced has been explored only in buffer systems which, although physiologically relevant, lack the many serum components present in the blood and may not represent the actual binding behavior inside the body.

The binding of antibody to the target drug in the presence of biological fluids is the event that is expected to provide the medical benefit for drug addiction. Under normal circumstances, antibody molecules are too large to cross the blood brain barrier and hence much of the drug remains in the peripheral blood circulation [11]. As soon as the drug is consumed, these antibodies must capture the drug within the peripheral circulation before it reaches the central nervous system in order to block its pharmacological effect. The amount of antibody-drug complex formed is based on the available quantities of drug specific antibodies in the serum and their ability to recognize the drug. The tightness of binding between the antibody and the drug is measured as the affinity or equilibrium dissociation constant (*K*_D_). Earlier, Paula et al. used radioimmunoassay (RIA) together with immunoprecipitation to determine the affinity of a mouse monoclonal antibody (2E2) using radiolabeled cocaine and a detailed specificity analysis with about 30 different cocaine analogs [12], [13]. However, this approach requires complicated radioactive labeling of the drug, and an additional precipitation step to separate the bound antibody. Later Meijler et al. developed a fluorescent analog of a cocaine hapten to characterize cocaine monoclonal antibodies using solution phase equilibrium dialysis bypassing the need for a radioactive tracer and a precipitation step [14]. However, these approaches were primarily used to measure the affinity of pure monoclonal antibodies in saline buffer and gave no information as to the influence of biological matrices (e.g. serum, cell lysate) and the thermodynamic nature of the binding. In addition, when serum samples were used, the non-specific binding of the radioactive tracer and its influence on the measured affinity were not available. Furthermore, in either approach since the probe is only on the cocaine or its hapten molecule, only a competitive assay is performed to study the interactions with other cocaine analogs or metabolites and not a direct binding assay. Hence, there is a constant need for a reliable assay to understand thermodynamic and kinetic interactions so as to support basic and clinical research. Fluorescence correlation spectroscopy (FCS) and fluorescence resonance energy transfer (FRET) methods have been developed, but these are best suited mostly for large complexes to monitor diffusion properties.

**Figure 1:**
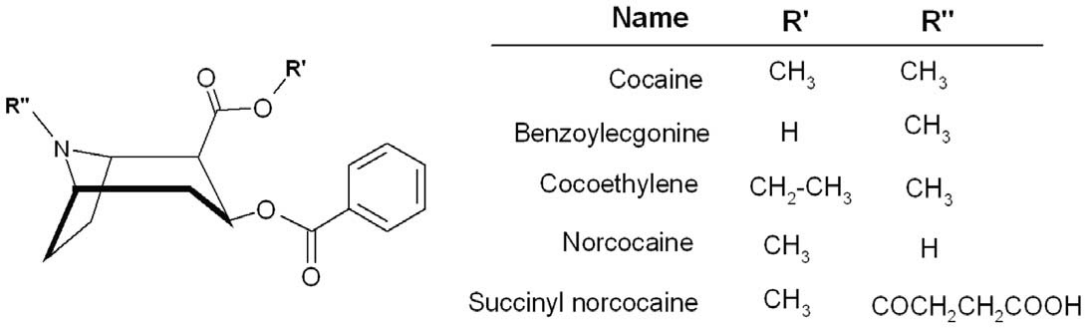
Schematic structure of cocaine and its common metabolites benzoylecgonine, cocaethylene, norcocaine.

ITC is a widely adopted, highly sensitive label-free technique for the measurement of equilibrium dissociation constants and the underlying thermodynamic parameters of complex formation by measuring the heat of a binding reaction [15]–[17]. SPR is a surface based method which enables characterization of both equilibrium and kinetic parameters of interactions. MST is a novel solution phase method capable of providing binding data for interactions by measuring the depletion of a fluorescently tagged binding partner in a microscopic temperature gradient in bulk solutions. A detailed description of this method can be found elsewhere [18], and it is a suitable approach compared to many other bioanalytical techniques that allow measuring of equilibrium dissociation constants in the presence of complex biological fluids [19]. Utilizing such an assay is likely to provide an opportunity to understand the affinity properties of antibodies produced by different vaccine formulations and obtained either from animals or patients and therefore closer to the *in vivo* condition. The above techniques provide an arsenal of methods to quantify and provide an understanding of the effect of different interactions between proteins and small molecules, e.g drugs with antibodies in the context of buffers solutions and major serum components such as serum albumin, immunoglobulin etc. The purpose of this study is to understand the equilibrium and kinetic binding properties and specificity of representative polyclonal antibodies relevant to cocaine immunotherapy in physiological buffer, and also to understand how the affinity changes in presence of serum. For this we first characterized a representative monoclonal antibody (mAb08) which binds to cocaine, benzoylecgonine (BE) and cocaethylene (CE) and extended the knowledge to address kinetic constants of polyclonal antibodies purified from mouse and human serum. We chose three human serum samples from patients who had high responses (∼110–120 µg/ml) to TA-CD (therapy for addiction- cocaine dependency) vaccine and one mouse serum (RR6 ∼450 µg/ml) from one of our cocaine vaccine formulations similar to TA-CD vaccine, but with a different carrier protein. These subjects were immunized using a succinylnorcocaine (SNC) hapten (Fig.1) conjugated to a large immunogenic carrier protein.

## Materials and Methods

Cocaine hydrochloride was obtained from National Institute of Drug Abuse (NIDA). Benzoylecgonine was generated by boiling cocaine in water overnight, cooling the resulting solution and collecting the precipitate. Cocaethylene and BSA-SNC were prepared using the method described by Everhart *et al* 1999 [20] and Hrafnkelsdottir *et al* 2005 [21], respectively. The purity of the compounds was checked by thin layer chromatography and NMR. The hapten density of BSA-SNC was determined using MALDI-TOF at the Laboratory for Biological Mass Spectrometry Texas A&M University College Station, Texas. The antibody mAb08 was purchased from US Biological (# B1077-08) and it was raised using a benzoylecgonine hapten coupled to keyhole hemocyanin (KLH) via substituting a linker in the methyl ester position of cocaine. The manufacturer states that the antibody binds to BE with > 100% cross reactivity to cocaine. The antibody mAb 402 was purchased from Arista Biologicals (ABCOC-402), and is recommended for drug testing assays.

### Isothermal Titration Calorimetry

ITC experiments were performed using an ITC-200 micro calorimeter from MicroCal (Northampton, MA, USA). Samples were dialyzed against PBS buffer and degassed prior to loading into the ITC cell. The drug samples were prepared in the same buffer to avoid buffer mismatch. Titrations were performed at 25°C by injecting consecutive aliquots (2 µl) of 45 µM drug into the ITC cell (volume of 200 µl) containing 3 µM antibody at 500 rpm stirring rate. Intervals between injections were set to be 150 sec in order to allow the heat from each injection to return to the baseline. Heats of dilution control experiments were performed by titrating ligand into buffer. The heats associated with the control titration were subtraced from the raw binding data prior to fitting. The data were fitted assuming that the antibody binding sites are identical using a single set binding model included in the Origin 7.0 software provided by the manufacturer. ITC directly measures the change in heat associated with two components going from the free to the bound state. Thus the observed molar change in enthalpy (ΔH_obs_) can be determined [22]. Furthermore the ΔH_obs_ provides a probe for the extent of ligand binding throughout a titration which allows the calculation of the *K*_A_. With these two terms and knowing the experimental temperature a complete thermodynamic characterization of an interaction based on an equation: ΔG_obs_  =  *−*RT×ln(*K*_D_)  =  ΔH_obs_ −TΔS_obs_, where R is the gas constant, T is the absolute temperature, and ΔG_obs_, ΔH_obs_, and ΔS_obs_ are the observed changes in standard free energy, enthalpy, and entropy on going from unbound to bound states, respectively.

### Microscale Thermophoresis (MST)

The fluorescent labeling was performed with a reactive RED dye (NT-647) following the manufacturer’s protocol (Nanotemper, Germany). The labeling procedures were performed on both the monoclonal antibody mAb08 and the immune conjugate (BSA-SNC), and the reaction conditions were optimized to give about 2 tracer molecules per protein as described by the manufacturer (Nanotemper, Germany). Free dye was removed by purification on a Sephadex G-25 column provided with the kit. MST assays were carried out as described in Jerabek-Willesmsen et al. [18]. Serial dilutions of unlabeled drug or antibody samples were mixed with 20 nM of NT-647-labeled proteins in PBS buffer (25 mM phosphate pH 7.4, 150 mM NaCl, 0.05% Tween-20) and incubated for 20 minutes. About 10 µl of sample was loaded into standard monolith NT capillaries provided by the manufacturer and assays were performed in a Nanotemper Monolith NT.015T. The Red fluorescence was exited using a light-emitting diode, and its emission was recorded at a focused location of capillary. Using another infrared laser diode a microscopic temperature gradient was created at the same location and the fluorescence depletion was measured. By plotting the concentrations of unlabeled binding partner with the changes in fluorescent thermophoresis signal, *K*_D_ values were determined using the Nanotemper analysis software. The overlay diagram was made by normalizing the data to fraction bound.

### Surface Plasmon Resonance (SPR)

SPR analyses were performed using BIAcore 3000 optical biosensors with research grade C1 sensor chips (BIAcore, Uppsala, Sweden). To reduce the avidity effect and molecular crowding at the chip surface, C1 chips were chosen over the regular carboxymethylated chips such as CM5 or CM4 to prepare low ligand surface immobilization. Since the BSA-SNC conjugates were synthesized by targeting the amino acid lysine of BSA, ligand-thiol coupling was performed targeting the cysteine residues of BSA-SNC. The chips were activated using 0.2 M *N*-ethyl-*N*-(dimethylaminopropyl) carbodiimide (EDC) and 0.05 M *N*-hydroxysuccinimide (NHS) at a flow rate of 10 µl/min, followed by injecting 40 µl of coupling agent 2-(2-pyridinyldithio) ethaneamine hydrochloride (PDEA) in pH 8.5 borate buffer for 4 minutes. To the activated surface, BSA-SNC (1 µg/µl) was injected for 7 minutes. The residual activated groups were quenched by blocking the surface with excess of cysteine-hydrochloride. Using similar conditions, unmodified pure BSA was immobilized at flow cell 1 as a reference surface.

Data was collected using a medium collection rate at 25°C. The instrument was primed a few times using the running buffer before injecting the concentration series. For every binding cycle, the antibody samples were injected for 1.5 minutes at 50 µl/min flow rate and dissociation was monitored for 5 minutes. The bound antibody was releasedwith two 15 µl injections of pH 1.5 glycine buffer. The kinetic constants of binding were obtained using a 1∶1 Langmuir binding model. The raw data sets were analyzed using BIAevaluation (version 3.2) provided by BIAcore Inc. (Uppsala, Sweden). After the subtraction of background responses from the control flow cells, the association and dissociation phases were globally fitted for all the sensorgrams simultaneously, omitting any noisy data at the beginning and end of the analyte injection. In the case of solution competitive experiments, a simple non-linear regression analysis was performed with log inhibitor concentration against the observed binding response using Graph-pad prism to determine the IC50 values.

## Results

### Antibody Affinity to Cocaine in Presence of Serum using MST Assay

**Figure 2:**
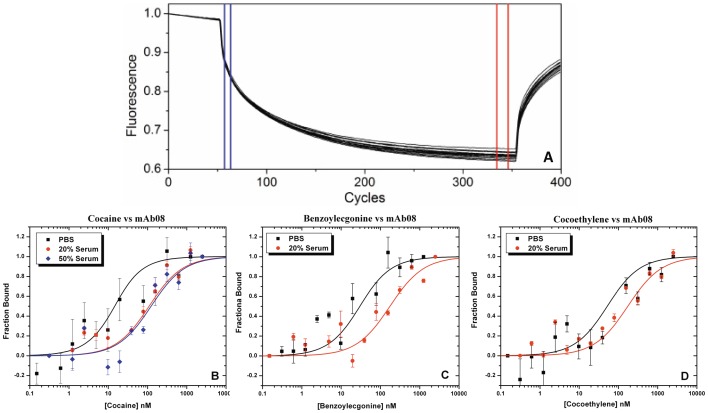
Thermophoresis raw data of *f*-mAb08 binding to different concentrations of cocaine from 1500 to sub-nanamolar range **(A)**. The blue and red lines are the region corresponds to cold and hot fluorescence thermophoresis, respectively. Normalized fraction bound calculated from the raw thermophoresis data of 20 nM *f*-mAb08 binding to **(B)** cocaine, **(C)** benzoylecgonine, and **(D)** cocaethylene in PBS buffer (black circles) and 20% (red circles) or 50% (blue diamonds) human serum.

MST analyses were performed by titrating 20 nM of fluorescent labeled *f*-mAb08 (∼1000 fluorescence counts) mixed with increasing concentrations of cocaine ranging from sub-nanomolar to 1 µM. The direct binding of native cocaine to the antibody was observed as a change in thermophoresis of the fluorescently labeled antibody upon complex formation. The decrease in the fluorescence counts of bound complex compared to unbound antibody with increasing concentration of drug indicates a positive thermophoretic effect as the *f*-mAb08 moves away from the laser source (Fig 2A). Fitting the thermophoretic data points, the ratio of the fluorescence counts between hot (red line) and cold (blue line) with different concentrations of cocaine gives a dissociation constant (*K*_D_) of 7.8 nM in PBS buffer (Fig 2B, black). Since MST can measure the interactions in complex serum, we titrated 20 nM of *f*-mAb08 in 20% control human serum with increasing concentrations of cocaine. Background intrinsic fluorescence from the serum proteins were excluded by linking a high wavelength red fluorescent tracer (*λ_Ext_*  = 647 nM) to mAb08. We noticed the *K*_D_ value was shifted from 7.8 nm to 105 nM, which is about 14 fold decrease in affinity. However, there was no further significant change in the affinity observed when serum concentration was increased from 20% to 50%. A similar trend was also noticed when titrating *f-*mAb08 with BE (Fig 2C) and CE (Fig 2D) with a *K*_d_ of 23.7 and 47 nM in PBS and 98 and 169 nM in 20% serum, respectively. The *K*_D_ values were reproducible with different sample preparations and also using the same antibody obtained from different batches. Such shifts are expected to rise in the presence of serum due to the competitive interactions with a large number of different biological molecules or change in viscosity. The capacity of the antibodies produced by the immune system to discriminate between similar molecular structures in a crowded biological matrix remains a fascinating achievement of nature.

### ITC of mAb08 Binding to Cocaine and Derivatives

**Figure 3:**
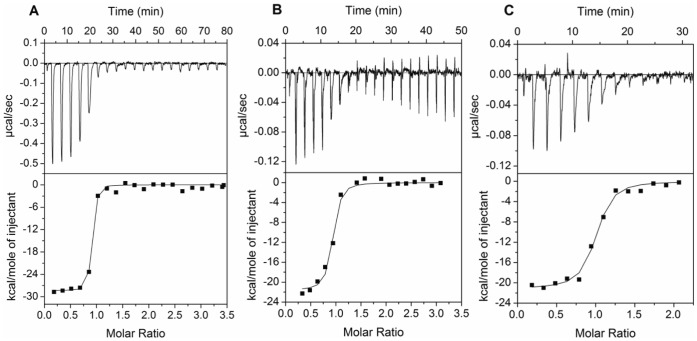
Calorimetric titrations of 3 µM mAb08 with 45 µM of (A) cocaine, (B) benzoyl-ecgonine, (C) cocaethylene. Top panel illustrates the raw data of ITC titrations of 20 equal injections of drug to 3 µM mAb08 antibody. Bottom panel illustrates the non-linear least squares fit of the peak area from the top panel to a binding model.

The thermodynamic properties of the binding of mAb08 to cocaine, benzoylecgonine, and cocaethylene were evaluated in pH 7.4 PBS buffer at 25°C using ITC. As shown in the upper panel of Fig. 3, the titration of 45 µM cocaine, BE, and CE against 3 µM mAb08 shows specific binding and formation of complexes with exothermic heat of binding. As the concentration of ligand increases in the cell the availability of free antibody binding sites decreases and hence lower the amount of exothermic heat till saturation is achieved. The binding isotherms, which is the incremental heat release as a function of complex formation is shown in the lower panel of Fig. 3. The solid line corresponds to the fit to a binding model to yield the equilibrium binding constant, *K*_D_, and the binding parameters, *n* and Δ*H*_obs_. The results of data analysis are shown in Table 1 along with the derived parameters, Δ*G_cal_* and Δ*S_cal_*. The affinities of the interactions of mAb08 to cocaine, BE, and CEare 2.5, 18.6, 34.4 nM, respectively (Table 1) resulting in ΔG_obs_ values in the range −11.7 to −10.5 kcal mol^−1^. The accompanying ΔH_obs_ values are large and favorable (ranging −28 to −21 kcal. mol^−1^. The ΔS_obs_ values are negative (ranging from −37.3 to −54.8 cal.mol^−1^ K^−1^). The interactions are all highly enthalpy driven suggesting that the net formation of non-covalent bonds is a major contributor to the affinity. In addition to the specific binding to cocaine, high affinity to pharmacologically active cocaethylene metabolite adds desirability to cocaine immunotherapy. However, high affinity to inactive benzoylecgonine, if present at high concentrations in the serum, is likely to reduce the amount of antibody available for native cocaine binding. Since mAb08 was elicited with a cocaine hapten conjugated in the methyl ester position, it is likely that conjugate vaccines linked in other positions (e.g., on the tropane ring using norcocaine) may have much less affinity for benzoylecgonine.

**Table 1:** Thermodynamic binding parameters of antibody binding to cocaine and its metabolites obtained from ITC titrations at 25°C.

Sample	Drug	N	ΔH_obs_ kcal mol^−1^	ΔS_cal_ cal.mol^−1^K^−1^	ΔG_cal_ kcal mol^−1^	*K*_D_ nM
mAb08	Cocaine	0.84	−28.08±0.37	−54.8	−11.7	2.5±6.8
mAb08	Benzoylecgonine	0.88	−21.66±0.61	−37.3	−10.5	18.6±48.7
mAb08	Cocaethylene	0.95	−21.16±0.48	−36.8	−10.2	34.4±129
mAb08+7 µM hIgG	Cocaine	0.83	−28.58±0.35	−57.4	−11.5	4.0±10.5
mAb08+20% Serum	Cocaine	0.91	−33.19±0.61	−79.5	−9.5	113±820
20% Serum	Cocaine	1.47	−21.82±3.16	−47.1	−7.8	2×10^3^±6.5×10^3^
Clone 402	Cocaine	1.0	−26.4±1.93	−65.6	−6.8	10×10^3^±80×10^3^

### Cocaine Interactions with Human Serum and Human Serum Albumin

**Figure 4:**
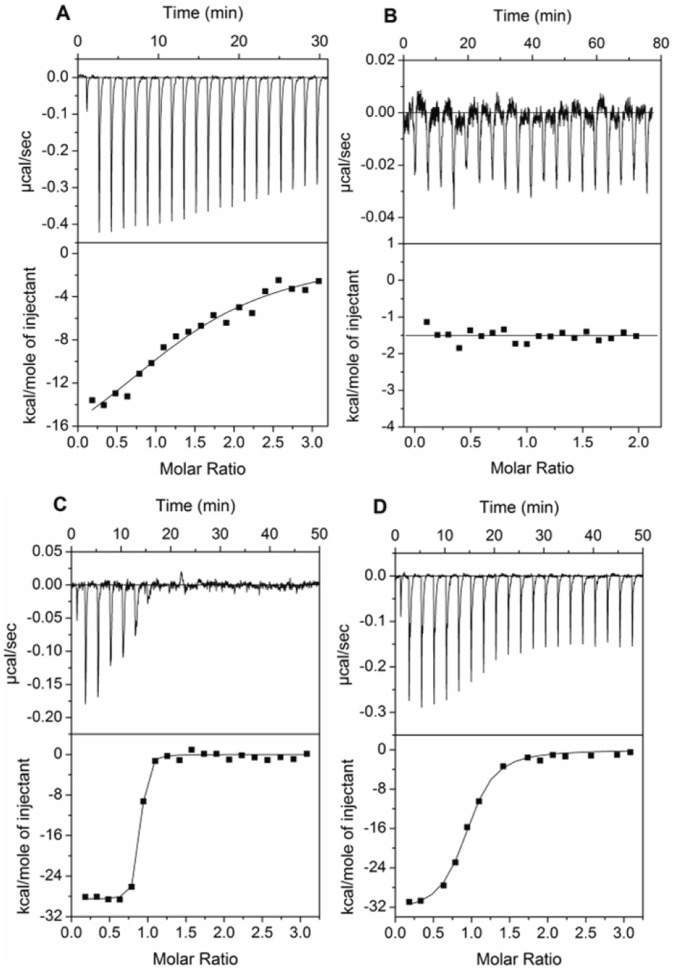
Calorimetric titrations of **(A)** 45 µM cocaine to 20% human serum. **(B)** 45 µM cocaine to 5 µM human serum albumin. **(C)** 3 µM mAb08 in 7 µM human polyclonal antibodies with 45 µM cocaine **(D)** 3 µM mAb08 in 20% human serum with 45 µM cocaine.

The thermodynamic parameters associated with the binding of cocaine to human serum and human serum albumin were investigated using ITC. We titrated 45 µM of cocaine to 20% of control human serum (no anti-cocaine antibodies) dialyzed against PBS (Figure 4A). Fitting the exothermic isotherm resulted in a K _D_ of 2 µM. Since human serum albumin (HSA) is a major carrier protein present in human serum, we titrated 5 µM of pure HSA against 45 µM of cocaine in PBS and found no detectable binding in that concentration range (Fig 4B) suggesting that the dominant binding was to other serum proteins, such as alpha acidic glycoprotein [23], [24]. Since the objective of this study is to investigate the antibody affinity to cocaine in presence of serum, we did not pursue further titrations with other serum proteins. Instead we mixed mAb08 with 7 µM of nonspecific human IgG (Pierce # 31879) and titrated against 45 µM cocaine (Fig 4C). Interestingly the presence of non-specific antibodies does not alter the calorimeteric properties of mAb08 significantly, with unaltered observed enthalpic value (ΔH_obs_  =  −28 kcal mol^−1^) as compared to titrations performed with pure mAb08 in PBS. However, the titration of mAb08 in the presence of 20% serum changed the calorimetric profile significantly (Fig. 4D). While we anticipate more non-specific contribution from 20% serum, those contributions were carefully subtracted in the control experiment and the binding constant derived from the fit is close to 113 nM, which is closer to the values obtained from the MST assay.

### Affinity of Polyclonal Antibodies using Solution Phase MST

While measurements of the thermodynamic and kinetic properties of monoclonal antibodies could be of significant importance and relevant to passive immunotherapy, the serum samples from active immunotherapy are polyclonal and have different levels of drug specific antibodies. This also adds another step of complication to purifying the specific antibodies from bulk serum in order to characterize them using different bioanalytical techniques. In addition, it is also not possible to covalently link a fluorophor to the drug specific antibody without performing affinity purification. We purified the enriched IgG fraction from serum using a Protein A/G affinity resin packed in a 1 ml Phytip column (Phynexus Technology, Inc.) as described by the manufacturer and concentrated it using 30 kDa centrifugal concentrators (GE Healthcare). To determine the dissociation constant of polyclonal antibodies using the MST approach, we have covalently linked a flurophore (NT-647) to BSA-SNC to mimic native cocaine. Similar immune conjugates are used to immunize animal or patients, and also used for ELISA screening assays to measure the immune response to cocaine.

**Figure 5:**
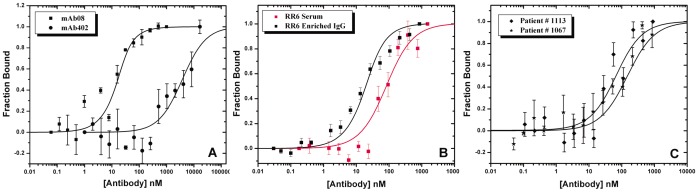
Normalized fraction bound calculated from the raw thermophoresis data of 20 nM *f*-BSA-SNC binding to different concentrations of (A) monoclonal antibodies mAb08 and mAb402, (B) vaccinated mouse serum (blue) and enriched IgG fraction, (C) enriched IgG fraction of vaccinated patient serum.

MST analysis of *f*-BSA-SNC was first validated by measuring the dissociation constant of monoclonal antibodies (mAb08 and clone 402) characterized using ITC. The binding of *f*-BSA-SNC to mAb08 and clone 402 showed positive thermophoresis similar to the data shown in Fig 2A. The change in the amplitude of thermophoresis of *f*-BSA-SNC with mAb08 was higher compared to the low affinity clone 402 antibody. As demonstrated in Fig. 5A, the normalized bound fraction of *f*-BSA-SNC to mAb08 and clone 402 showed *K*_D_ values of 7.8 nM and 10 µM, respectively, which indeed were close to the values obtained from ITC titration of cocaine against respective antibodies.

To determine the *K*_D_ of polyclonal antibodies, we used a mouse serum pool (RR6) which was obtained following vaccination with one of our cocaine vaccine formulations (*unpublished results*). This pool was chosen based on the high antibody titer values (442 µg/ml or 3000 nM as determined by ELISA), which were easier to make several dilutions starting from 1500 to 0.05 nM. As shown in Fig 5B, 20 nM of *f*-BSA-SNC was titrated against enriched IgG or with 50% RR6 serum from 2000 to 0.01 nM concentration. Fitting the data points provided a *K*_D_ value of 9.7 nM for the enriched fraction, which in presence of 50% RR6 serum shifted to 73 nM (∼8 fold decrease). It is interesting to see that this 8 fold decrease is less than the 14 fold decrease noticed when *f*-mAb08 was titrated against cocaine. Though this difference is less, BSA-SNC may have a different nonspecific interaction to the serum as compared to free cocaine.

Furthermore, we were interested in utilizing this method to measure the *K*_D_ value of polyclonal antibodies produced by patients who were vaccinated with succinylnorcocaine- cholera toxin B-subunit protein, the TA-CD- vaccine [9]. We took serum samples from two patients with high responses to the vaccine, whose antibody levels were in the range of 115 µg/ml. Enriched IgG fractions were purified from the sera and the MST assays were performed at several dilutions from 900 to 0.03 nM with 20 nM of *f*-BSA-SNC (Figure 5C and Table 2) and the *K*_D_ values determined for the patients # 1113 and 1067 were 57 and 139 nM, respectively. Patient 1113 markedly reduced his cocaine use during the period that antibody levels were high, suggesting clinical efficacy in the presence of higher affinity antibodies, supporting the central hypothesis of needing high affinity antibodies for successful development of cocaine immunotherapy.

**Table 2:** Monoclonal and polyclonal antibody binding affinity (*K*_D_) obtained from MST assay at 25°C.

Sample	Drug	*K*_D_^1^ (nM) Enriched IgG	*K*_D_^2^ (nM) (Serum%)
*f*-mAb08	Cocaine	7.8	105 (20%)
*f*-mAb08	Cocaine	7.8	128 (50%)
*f*-mAb08	Benzoylecgonine	23.7	98 (20%)
*f*-mAb08	Cocaethylene	47	169 (20%)
mAb08	*f*-BSA-SNC	6.6	-
Clone402	*f*-BSA-SNC	3778	Too weak
RR6	*f*-BSA-SNC	9.7	73 (50%)
TA-CD #1067	*f*-BSA-SNC	139	-
TA-CD #1113	*f*-BSA-SNC	57	-

BSA-SNC with low hapten density (7 SNC per BSA determined by MALDI TOF) was further modified with NT-647 flurophore. *K*_D_^1^ and *K*_D_^2^ are the binding affinity obtained using MST assay in presence of phosphate buffer and 20 or 50% control human serum, respectively.

### Kinetics and Inhibitory Binding Constants of Antibodies using Surface Plasmon Resonance Studies

Antibody binding kinetics were typically studied using surface plasmon resonance studies by covalently immobilizing the antibody on the chip surface and injecting the antigen as an analyte. However, this approach did not work well with cocaine. Being a small molecule, the binding responses of cocaine were low and also the partial positive charge on the cocaine non-specifically bound to the modified or unmodified negatively charged dextran at the chip surface. Increasing the ionic strength and/or changing the surfactant (tween) concentration or adding bovine serum albumin to the buffer also did not help to resolve this problem. In addition, our attempts at using dextran free C1 chips were also unsuccessful to immobilize sufficient high density of antibodies to get a reasonable cocaine binding response. This led us to immobilize BSA-SNC on the chip surface and injecting antibody as an analyte. Avidity effects were cautiously reduced by minimizing the number of succinyl-norcocaine (SNC) molecules linked to bovine serum albumin (BSA) and by immobilizing a low density (50 Ru) of BSA-SNC on the chip surface without dextran matrix (C1). Antibodies of different concentrations were injected at a high 50 µl/min flow rate to avoid potential mass transport effects and the bound antibodies were removed with pH 1.5 glycine buffer.

**Figure 6:**
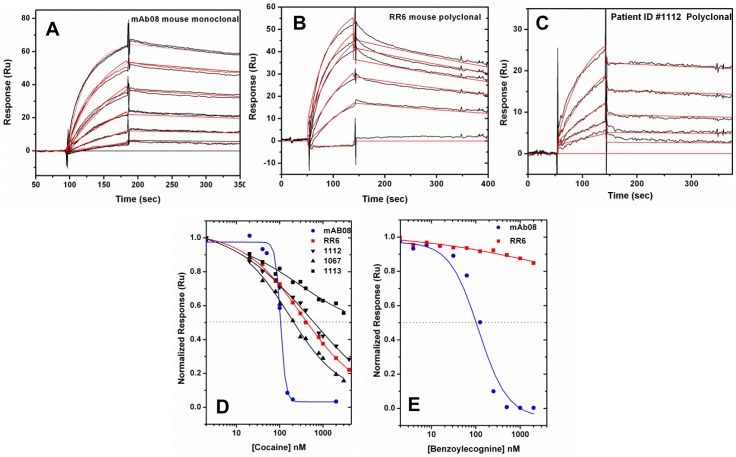
SPR kinetic analysis of antibody binding to immobilized BSA-SNC at 25°C. Two fold dilution of **(A)** mAb08 (100 to 0 nM), **(B)** RR6 mice enriched polyclonal antibody, (50 to 0 nM), **(C)** vaccinated patient (TACD # 1112) enriched polyclonal antibody. (50 to 0 nM) was injected for 1.5 minutes at 50 µl/min flow rate over the C1 chip, which was immobilized with BSA-SNC using surface thiol coupling at a low density of 50 Ru. The black lines are the different sensorgrams obtained from BIAcore3000, and the red lines are the theoretical global fit to a 1∶1 interaction model. Panel **(D)** and **(E)** are the competitive inhibition of different antibody binding to the immobilized BSA-SNC with increasing concentration of cocaine and benzoylecgonine, respectively.

Panel A of Figure 6 depicts the binding kinetics of the mAb08 to immobilized BSA-SNC from 100 to 0 nM concentrations with two-fold serial dilutions. The sensorgrams were replicated at all concentrations studied and each curve represents a separate cycle in which the antibody bound to the sensor surface was completely regenerated at the end of each cycle. The red lines in the figure represent a global fit of response data to a 1∶1 interaction model using the BIAevaluation software. The affinity constant (*K*D) of 1.87 nM obtained from the fit was very close to the value determined by isothermal titration calorimetry. The calculated *k*_a_ and *k*_d_ rates were 3.66×10^5^ (M^−1^s^−1^) and 6.85×10^−4^ (s^−1^), respectively (Table 3). Figure 6 also depicts the binding kinetics of enriched polyclonal antibody of RR6 mouse (Panel B) and patient antibody TA-CD # 1112 (Panel C) binding to immobilized BSA-SNC from 50 to 0 nM concentrations with two-fold dilutions (Table 3). The polyclonal antibodies exhibited slightly different interaction kinetics with an higher *k*_a_ as compared to mAb08. Such a high *k*_a_ may still be influenced by mass transport limitation and avidity effects, but they are likely to be largely reduced as described above. In addition, the estimated affinity of 2 nM from SPR for mAb08 matches very closely the value obtained from ITC measurements and MST assay. Similarly, in case of RR6, 1 nM affinity from SPR matches closely the value of 10 nM obtained from MST assay. Despite the close match of SPR affinity parameters to the values obtained from solution based techniques, more data is needed from low affinity antibodies to understand the avidity contributions. Even though it is a polyclonal antibody, the antigen recognition sites for these antibodies are small (cocaine molecular weight 303 grams/mole) and they are different from typical protein antigens which show multiple epitope binding sites.

**Table 3:** Kinetic rate constants of antibody binding to BSA-SNC obtained from SPR measurements.

Antibody	*k*_a_×10^5^ (M^−1^S^−1^)	*k*_d_×10^−3^ (s^−1^)	*K*_A_×10^9^ (M^−1^)	*K*_D_×10^−9^ (M)	_χ_^2^
Mouse mAb08	3.66	0.665	0.535	1.87	1.5
Mouse polyclonal RR6	12. 7	1.22	1.05	0.954	1.33
Human TA-CD # 1112	12.9	0.284	4.54	0.22	0.228

Competitive inhibition studies of mAb08 binding to BSA-SNC were performed by mixing 50 nM mAb08 with increasing concentrations of cocaine at a flow rate of 50 µl/min. As shown in panel D of Figure 6, the antibody binding response to BSA-SNC at the chip surface clearly decreased with increasing concentrations of cocaine. At high cocaine concentrations most of the antibodies were already bound to the cocaine present in the bulk solution phase and hence not available for binding to BSA-SNC. A sigmoidal dose response inhibition fit of these data provided an IC_50_ value of about 100 nM, which is ∼100 fold lower than the true equilibrium binding constant. The IC_50_ values were highly reproducible even at different antibody concentrations (50 or 100 nM) and also with different density levels of immobilized BSA-SNC at the chip surface. Similar experiments were also performed with enriched polyclonal antibodies purified from mouse or patient serum, and the results clearly demonstrated the specificity to native cocaine with different IC_50_ values from 100 to 500 nM. Similar competitive antibody binding experiments were also performed with increasing concentrations of BE for mAb08 and RR6 enriched polyclonal antibodies (Fig 6.E). Interestingly, mAb08 also showed specific inhibition to benzoylecgonine with an IC_50_ of 123 nM. Whereas the RR6 enriched fraction did not show any significant inhibition by benzoylecgonine.

## Discussion

Our group is actively involved in developing cocaine vaccines and we are currently conducting a phase IIb clinical trial using TA-CD vaccine. The goal of the TA-CD vaccine study is to negate the effects of cocaine by stimulating the human immune system to produce a high level of cocaine specific antibodies, preferably having high affinity so as to capture the pharmacologically active cocaine and cocaethylene, but not the inactive benzoylecgonine [1]. The efficacy of this approach will be determined by the qualitative and quantitative properties of the specific antibodies produced by the immune system. Theoretically this can be represented by a defined amount of antibody mixed with cocaine (or drug) in a closed compartment, in which the formation of antibody-drug complex and its dissociation are in constant equilibrium based on the qualitative nature of antibody to the drug. Based on the law of mass action, the interaction of a drug with a single antibody combining site (assuming the two binding sites of antibodies are identical) follows the equation as given below, where [Ab]*_free_*, [D]*_free,_* and [Ab-D]*_bound_* correspond to the free concentrations of drug specific antibody, free drug and bound complexes, respectively. The constants *k*_a_, *k*_d_ are the rates of complex formation and dissociation.



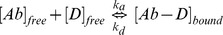
Since each of these components are in the same compartment, simple differential equations can be written to quantify each component with respect to time.



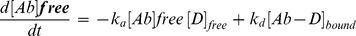
.



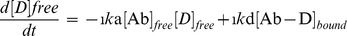
.



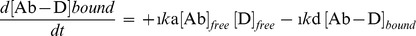
.



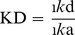
.

The above equations can be simulated to provide a snapshot of antibody-antigen interactions in real time, which will be useful to address the challenges associated with vaccine development in terms of the required quantitative and qualitative properties of antibodies. However, the usefulness of this binding model depends on determining the critical kinetic parameters (*k*_a_ and *k*_d_) of antibody-drug interactions, and the precise quantification of drug specific antibodies. Furthermore, it is important to understand that this as a simplified *in vitro* model, with the assumption that all the components are in the same container throughout the simulation period without any degradation. This gets complicated and different in live rodents and humans based on the ADME (adsorption, distribution, metabolism and elimination) properties of cocaine. While the rate and relative amount of cocaine entering the peripheral circulation depend greatly on the route of administration with a elimination half-life ranging from 60 to 90 min, it degrades to inactive BE by endogenous enzymes in tissue or by nonenzymatic hydrolysis at increased pH, or slowly to ecgonine and benzoic acid in circulation from the action of butyrylcholinesterase. The degraded BE has a longer elimination half-life from 6–8 hrs and its plasma concentration fluctuates in cocaine addicts and often get higher than the cocaine concentration [25], [26]. Therefore, a strong binding preference for cocaine will be required for efficient active drug binding by antibodies.

**Figure 7:**
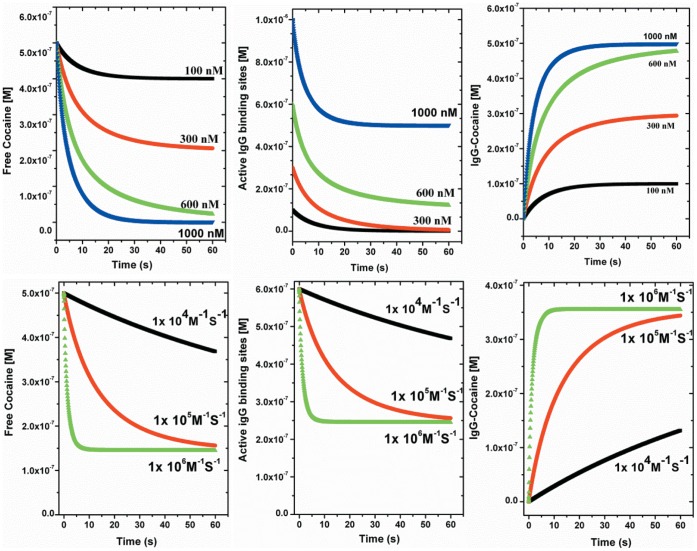
Simulated curves based on the binding model discussed. Starting from the left, the panels shows the simulated concentration of [Cocaine]*_free_*, [Anti IgG binding sites]*_free_*, and [IgG-Cocaine]*_bound_*. The top three panels were simulated using the parameters [Cocaine]*_free_*  = 500 nM, *k*_a_  = 3.66×10^5^ M^−1^S^−1^, *K*_D_  = 1.87 nM and at different concentrations of [Anti IgG binding sites]*_free_*  = 100, 300, 500, 600 and 1000 nM. The lower three panels were simulated using the parameters [Cocaine]*_free_*  = 500 nM, [Anti IgG binding sites]*_free_*  = 600 nM (which is half of actual IgG concentration), *K*_D_  = 100 nM and at different *k*_a_  = 1×10^4^, 1×10^5^, 1×10^6^ M^−1^S^−1^_._

In earlier studies pharmacological effects were noticed within 2 minutes with peak plasma concentrations of cocaine in the range of 150–500 nM in patients who smoked 10–40 mg of cocaine base [27]. For an equimolar amount of antibody binding site concentration, a minimum of at least 250 nM (∼40 µg/ml) of drug specific IgG antibody must be present in the plasma. To demonstrate binding kinetics in this model (top panel of Figure 7), simulations were run from 0 to 60 seconds using the kinetic parameters obtained for mAb08 in PBS (*K*_D_  = 1.87 nM; *k*_a_  = 3.66×10^5^ M^−1^S^−1^; [Cocaine]*_free_*  = 500 nM) at different binding site concentrations. IgG molecules have 2 binding sites that interact with small molecules independently of each other. The curves show that the initial 500 nM of [Cocaine]*_free_* drops very rapidly, depending on the concentration of available binding sites. At the 600 nM concentration about 80% of initial free cocaine is bound to the antibody within ∼20 seconds. However, at concentrations of 100 and 300 nM much of the cocaine remains unbound, which could cross the blood brain barrier and stimulate the pharmacological effects. In addition, the simulation of the concentration of free active binding sites shows that, at the end of 60 seconds, there are still some free IgG binding sites available at 600 nM and much more available at 1000 nM. Achieving such high concentrations (∼75 µg/ml) of specific IgG with similar kinetic parameters would be highly beneficial to optimize vaccination benefits for clinical addiction therapy.

In this model, we have assumed that the antibody binding sites are 100% functional and each antibody molecule binds to two drug molecules. However, the stoichiometric ratio (*N*) obtained from the ITC titration of mAb08 to the drugs are only closer to one drug per antibody molecule, instead of two (See Table 1). This may arise from antibody aggregation or other post translational modification such as oxidation, deamidation of amino acids in the drug binding sites of antibody. Furthermore, similar issues were noticed recently with a significant reduction in antibody binding capacity while evaluating the efficacy of antimethamphetamine monoclonal antibody in rats [28].

Besides the importance of the concentration of specific antibody, the ability of antibody to sequester the drug in the presence of complex serum and the interaction of antibody as well as the drugs to the serum components are critical. MST analysis of 20 nM *f-*mAb08 binding to native cocaine in PBS demonstrates that the affinity constant is very close to the value obtained from ITC measurements. In addition, it also shows a fourteen fold decrease in the effective affinity of *f-*mAb08 to cocaine in the presence of 20% human serum, but with no further significant change observed in 50% human serum. Similar results were observed by Wienken, et al., with a 17 fold decrease in affinity when human interferon gamma antibody binding was studied in crude *E.coli* cell lysate, and a 400 fold decrease with a quercetin inhibitor binding to cAMP kinase in presence of 30% human serum [19]. Drugs such as quercetin and digitonin which bind to HSA strongly are likely to alter the effective affinity of their specific antibody or enzyme in presence of serum. Although cocaine does not bind strongly to HSA (see Fig 4B), other proteins, such as alpha-1-acidic glycoprotein [23], [24] may underlie the weak binding in 20% serum. This binding could have a significant impact on low affinity antibodies such as clone 402 (Arista Biologicals), which has only about 10 µM affinity to cocaine. Furthermore, the unaltered binding isotherm of mAb08 in the presence of 7 µM non-specific human IgG (Fig 4C), demonstrates the absence of non-specific cocaine binding to human IgG, and thus it may be useful to perform a direct ITC titration of enriched polyclonal antibodies against cocaine or other drugs.

Based on the MST assays on mAb08 in presence of 50% serum the affinity reduced to about 100 nM. The simulations were performed with *K*_D_  = 100 nM; [Cocaine]*_free_*  = 500 nM; [Active IgG binding sites]*_free_*  = 600 nM; at different association rate constants *k*_a_ from 1×10^4^–1×10^6^ M^−1^S^−1^. As shown in the lower panel of Figure 7, the simulated curves [Cocaine]*_free_* could only be suppressed from 500 nM to 150 nM even with *k*_a_ of 1×10^6^ M^−1^S^−1^. It is also interesting to look at the simulation of free active binding sites, at the end of 60 seconds more unbound antibody binding sites are available (∼200 nM) despite the presence of free cocaine (150 nM). However, changing the affinity from 100 to 1 nM, brings the concentration of [Cocaine]*_free_* closer to zero. Thus for a better clinical efficacy the stimulated polyclonal antibodies need to have a high average affinity in plausible quantities to sequester more circulating drug as soon as it is consumed, but before it reaches the brain. The binding model enables one to calculate how free drug must be suppressed by the antibody, leading to improvement in the clinical signs and symptoms of the disease.

RR6 is a mouse serum pool collected from group of mice vaccinated with one of our cocaine vaccine formulations, which produced high antibody titer and showed improved behavioral tests (Orson et al. unpublished results). Since the serum had high levels of specific antibodies, serial dilutions were made from 1500 nM to subnanomolar range directly with a control serum and mixed with 20 nM *f*-BSA-SNC. The apparent average dissociation constant determined from MST analysis was 73 nM for the antiserum, and 9.7 nM for the RR6 enriched IgG fraction in PBS buffer (Fig. 5B). The higher affinity also correlates to the improved behavioral tests in these vaccinated mice. Since the representative TA-CD patient serum antibody levels were substantially lower compared to the above RR6 serum level, we were unable to measure the thermophoresis of human antiserum samples. However, the enriched fractions from different patient sera showed *K*_D_ values in the range of 57–139 nM. The results suggest that the average affinities of RR6 mouse polyclonal antibodies are comparable to the mouse mAb08 both in the presence and absence of serum components. However, the average affinities observed with different human samples were about 6 and 14 fold lower compared to the mouse antibodies.

The SPR competitive inhibition studies of mA08 binding to the immobilized BSA-SNC shows a specific inhibition for cocaine with IC_50_ ∼104 nM and for BE with IC_50_ ∼123 nM. However, the polyclonal antibody RR6 shows a strong preferential specificity to cocaine compared to BE. Unfortunately due to limitation of human serum samples, we were unable to study the specificity to BE. However, this observation is consistent to our earlier inhibition studies on serum from other humans and mice vaccinated using similar SNC haptens [1]. Such lower specificity to BE will be of advantage in cocaine vaccine development, since binding to BE would otherwise reduce the amount of antibody available for cocaine binding.

In summary, in the present study we have shown the advantages of multiple biophysical approaches in studying cocaine-antibody interactions, and discussed their usefulness in optimizing our vaccine goals. As demonstrated here, ITC provides a true solution based affinity and thermodynamic characterizations of mAb binding to various soluble drug targets in saline buffer. However its applicability for polyclonal antibodies depends on the amount of serum and the quality of antibody purification. While the SPR competitive inhibition studies provide consistent specificity details, to validate the kinetic rate constants and avidity contributions more data is needed from low affinity antibodies. Thermophoresis analysis of monoclonal and polyclonal antibodies binding to cocaine reveals an approximately 15 and 8 fold reduction of affinity in complex serum over the purified antibody, respectively. The knowledge gained from this study will be helpful in comparing the responses to different vaccine constructs as well as the responses in different individual patients. In future studies, we plan to develop an improved fluorescent cocaine analog, and design a competitive inhibition assay to measure the affinity of antibodies from low or medium responders to better understand the clinical efficacy as related to the qualitative properties of the endogenous antibodies. The methods adopted based on MST and ITC analysis provide a novel approach to investigate antibody-small molecule interactions giving a thorough assessment of both thermodynamic and kinetic parameters.

## References

[1] Orson FM, Kinsey BM, Singh RA, Wu Y, Kosten TR (2009). Vaccines for cocaine abuse.. Hum Vaccin.

[2] Orson FM, Kinsey BM, Singh RA, Wu Y, Gardner T (2008). Substance abuse vaccines.. Ann N Y Acad Sci.

[3] Chi KR (2011). Vaccines move forward against a range of addictions.. Nat Med.

[4] Kinsey BM, Kosten TR, Orson FM (2010). Anti-cocaine vaccine development.. Expert Rev Vaccines.

[5] Fox BS, Kantak KM, Edwards MA, Black KM, Bollinger BK (1996). Efficacy of a therapeutic cocaine vaccine in rodent models.. Nat Med.

[6] Norman AB, Norman MK, Buesing WR, Tabet MR, Tsibulsky VL (2009). The effect of a chimeric human/murine anti-cocaine monoclonal antibody on cocaine self-administration in rats.. J Pharmacol Exp Ther.

[7] Norman AB, Tabet MR, Norman MK, Buesing WR, Pesce AJ (2007). A chimeric human/murine anticocaine monoclonal antibody inhibits the distribution of cocaine to the brain in mice.. J Pharmacol Exp Ther.

[8] Hicks MJ, De BP, Rosenberg JB, Davidson JT, Moreno AY (2011). Cocaine analog coupled to disrupted adenovirus: a vaccine strategy to evoke high-titer immunity against addictive drugs.. Mol Ther.

[9] Martell BA, Orson FM, Poling J, Mitchell E, Rossen RD (2009). Cocaine vaccine for the treatment of cocaine dependence in methadone-maintained patients: a randomized, double-blind, placebo-controlled efficacy trial.. Arch Gen Psychiatry.

[10] Carrera MR, Ashley JA, Parsons LH, Wirsching P, Koob GF (1995). Suppression of psychoactive effects of cocaine by active immunization.. Nature.

[11] Pardridge WM (2010). Biopharmaceutical drug targeting to the brain.. J Drug Target.

[12] Paula S, Tabet MR, Farr CD, Norman AB, Ball WJ (2004). Three-dimensional quantitative structure-activity relationship modeling of cocaine binding by a novel human monoclonal antibody.. J Med Chem.

[13] Paula S, Tabet MR, Keenan SM, Welsh WJ, Ball WJ (2003). Three-dimensional structure-activity relationship modeling of cocaine binding to two monoclonal antibodies by comparative molecular field analysis.. J Mol Biol.

[14] Meijler MM, Kaufmann GF, Qi L, Mee JM, Coyle AR (2005). Fluorescent cocaine probes: a tool for the selection and engineering of therapeutic antibodies.. J Am Chem Soc.

[15] Ladbury JE, Klebe G, Freire E (2010). Adding calorimetric data to decision making in lead discovery: a hot tip.. Nat Rev Drug Discov.

[16] Ladbury JE (2010). Calorimetry as a tool for understanding biomolecular interactions and an aid to drug design.. Biochem Soc Trans.

[17] Livingstone JR (1996). Antibody characterization by isothermal titration calorimetry.. Nature.

[18] Jerabek-Willemsen M, Wienken CJ, Braun D, Baaske P, Duhr S (2011). Molecular interaction studies using microscale thermophoresis.. Assay Drug Dev Technol.

[19] Wienken CJ, Baaske P, Rothbauer U, Braun D, Duhr S (2010). Protein-binding assays in biological liquids using microscale thermophoresis.. Nat Commun.

[20] Everhart ET, Jacob P, Mendelson J, Jones RT (1999). The synthesis of deuterium-labelled cocaine, cocaethylene and metabolites.. Journal of Labelled Compounds and Radiopharmaceuticals.

[21] Hrafnkelsdottir K, Valgeirsson J, Gizurarson S (2005). Induction of protective and specific antibodies against cocaine by intranasal immunisation using a glyceride adjuvant.. Biol Pharm Bull.

[22] Ladbury JE (1995). Counting the calories to stay in the groove.. Structure.

[23] Edwards DJ, Bowles SK (1988). Protein binding of cocaine in human serum.. Pharm Res.

[24] Parker RB, Williams CL, Laizure SC, Lima JJ (1995). Factors affecting serum protein binding of cocaine in humans.. J Pharmacol Exp Ther.

[25] Mets B, Diaz J, Soo E, Jamdar S (1999). Cocaine, norcocaine, ecgonine methylester and benzoylecgonine pharmacokinetics in the rat.. Life Sci.

[26] Lau CE, Ma F, Foster DM, Falk JL (1999). Pharmacokinetic-pharmacodynamic modeling of the psychomotor stimulant effect of cocaine after intravenous administration: timing performance deficits.. J Pharmacol Exp Ther.

[27] Jenkins AJ, Keenan RM, Henningfield JE, Cone EJ (2002). Correlation between pharmacological effects and plasma cocaine concentrations after smoked administration.. J Anal Toxicol.

[28] Atchley WT, Peterson EC, West CM, Owens SM (2011). Determining the Biochemical Basis of In Vivo Functional Changes to a Therapeutic Anti-Methamphetamine Monoclonal antibody using a Recombinantly Engineered IgG. National Biotechnology Conference. Hilton Union Square San Francisco, USA.. : American Association of Pharmaceutical Science.

